# *Talaromyces marneffei* infection associated with bronchiolitis obliterans in an HIV-negative child: a case report

**DOI:** 10.1186/s12879-022-07391-6

**Published:** 2022-05-16

**Authors:** Lin Lin, Huifeng Fan, Dongwei Zhang, Gen Lu

**Affiliations:** Department of Respiration, Guangzhou Women and Children’s Medical Centre, Guangzhou Medical University, No. 9, Jinsui Road, Zhujiang New City, Tianhe, Guangzhou 510120 Guangdong, China

**Keywords:** *Talaromyces marneffei*, Child, Sequelae, Bronchiolitis obliterans

## Abstract

**Background:**

*Talaromyces marneffei* is an opportunistic pathogen that infects immunodeficient and immunocompromised patients. We presented a pediatric patient with a diagnosis of *T. marneffei* infection who was followed up in the Guangzhou Women and Children’s Medical Centre.

**Case presentation:**

The child was a 5-year-old girl with persistent cough and gasping over 2 months who was confirmed with *T. marneffei* infection by bronchoalveolar lavage fluid culture and high-throughput sequencing technology. Human immunodeficiency virus (HIV) was negative according to a serum-specific antibody test. She was treated with amphotericin B and itraconazole as antifungal agents, with good clinical response. At follow-up, high-resolution computed tomography showed a mosaic sign in the whole lung field with a diagnosis of post-infectious bronchiolitis obliterans (PIBO) as the sequela. She has a mutated *COPA* gene with uncertain pathogenic potential on whole-exome sequencing.

**Conclusions:**

Clinicians should consider PIBO as a possible sequela in an HIV-negative paediatric patient with *T. marneffei* infection.

## Background

*Talaromyces marneffei* (formerly known as *Penicillium marneffei*) is an important dimorphic fungus. It is the only member in the genus that causes systemic mycosis and is more prevalent in South Asia [1]. In adults, *T. marneffei* infection has been considered to be exclusively associated with acquired immunodeficiency syndrome (AIDS) caused by human immunodeficiency virus (HIV) infection [2], although nowadays the infection rate in non-HIV-infected children has gradually grown [3], paediatric patients with primary immunodeficiency diseases (PIDs) being more susceptible according to previous reports [4, 5]. Here we report a rare case of post-infectious bronchiolitis obliterans (PIBO) as sequela after *T. marneffei* infection with a mutation in the *COPA* gene.

## Case presentation

In January 2019, a 5-year-old girl was hospitalized with intermittent fever, cough and shortness of breath for two months and she had recurrent lower respiratory tract infection from infancy. There was no family history of PIDs and consanguineous marriage. On admission, she had difficulty breathing. Stridor and moist rales were revealed by auscultation. Rash, lymphadenopathy, and hepatosplenomegaly were all absent. HIV was negative according to a serum-specific antibody test and HIV viral load. Humoral immunoassay showed decreased serum immunoglobulin G (IgG), IgA and IgM, but the serum IgE level was normal. Lymphocyte counts were all in their normal range on admission, including CD4^+^ subsets, CD8^+^ subsets, natural killer (NK) cells and CD19^+^ subsets. The nitroblue tetrazolium test (NBT) was normal (Table 1).

**Table 1:** Laboratory findings of the patient on the day of admission

Laboratory index	Result	Reference range
WBC (×109/L)	18.6	5–12
N (×109/L)	13.02	2–7.2
Hb (g/dL)	120.00	105–145
PLT (×109/L)	466.00	140–440
CRP (mg/L)	11.21	< 8.2
IgG (g/L)	4.56	5.0-10.6
IgA (g/L)	0.11	0.34–1.38
IgM (g/L)	0.16	0.44–1.44
IgE (IU/mL)	5.00	0–60.0
CD3 + 4+ (cells/µL)	1223.43	345–2350
CD3 + 8+ (cells/µL)	382.35	314–2080
CD19+ (cells/µL)	309.21	240–1317
Th/Ts (%)	2.02	0.47–2.05
NK (cells/µL)	285.62	210–1514

*WBC* white blood count, *N* neutrophils, *Hb* hemoglobins, *PLT* platelet count, *CRP* C-reactive protein, *Th* helper T cells, *Ts* inhibited T cells, *NK* natural killer

**Fig. 1:**
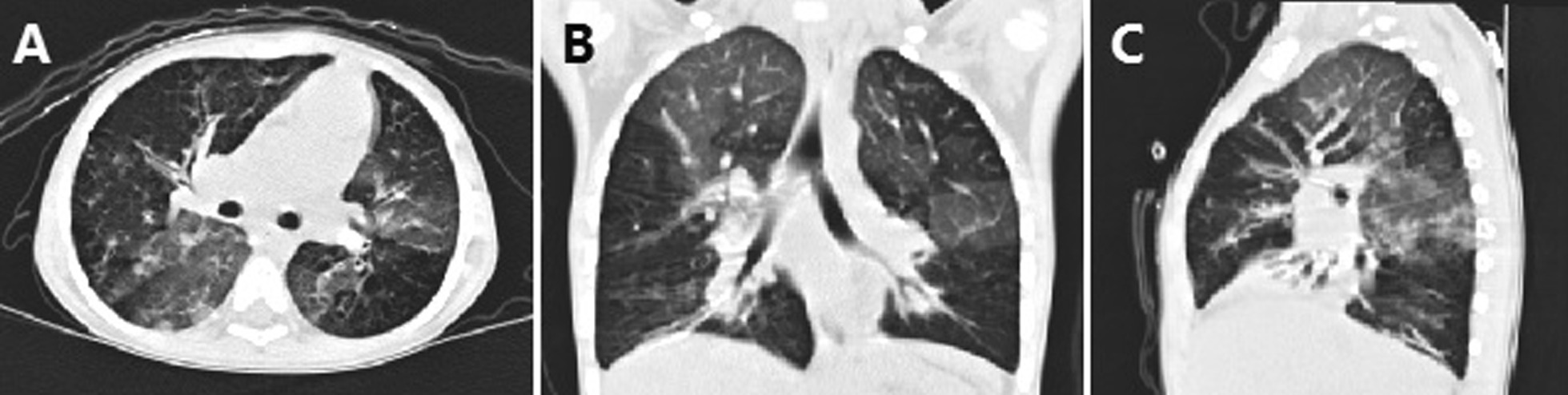
High-resolution computed tomography (CT) scan of the chest revealing small airway obstruction lesions with double pneumonia, insufficiency of the right middle and left lower lung segments, and local bronchiectasis in both lungs on first hospitalization

**Fig. 2:**
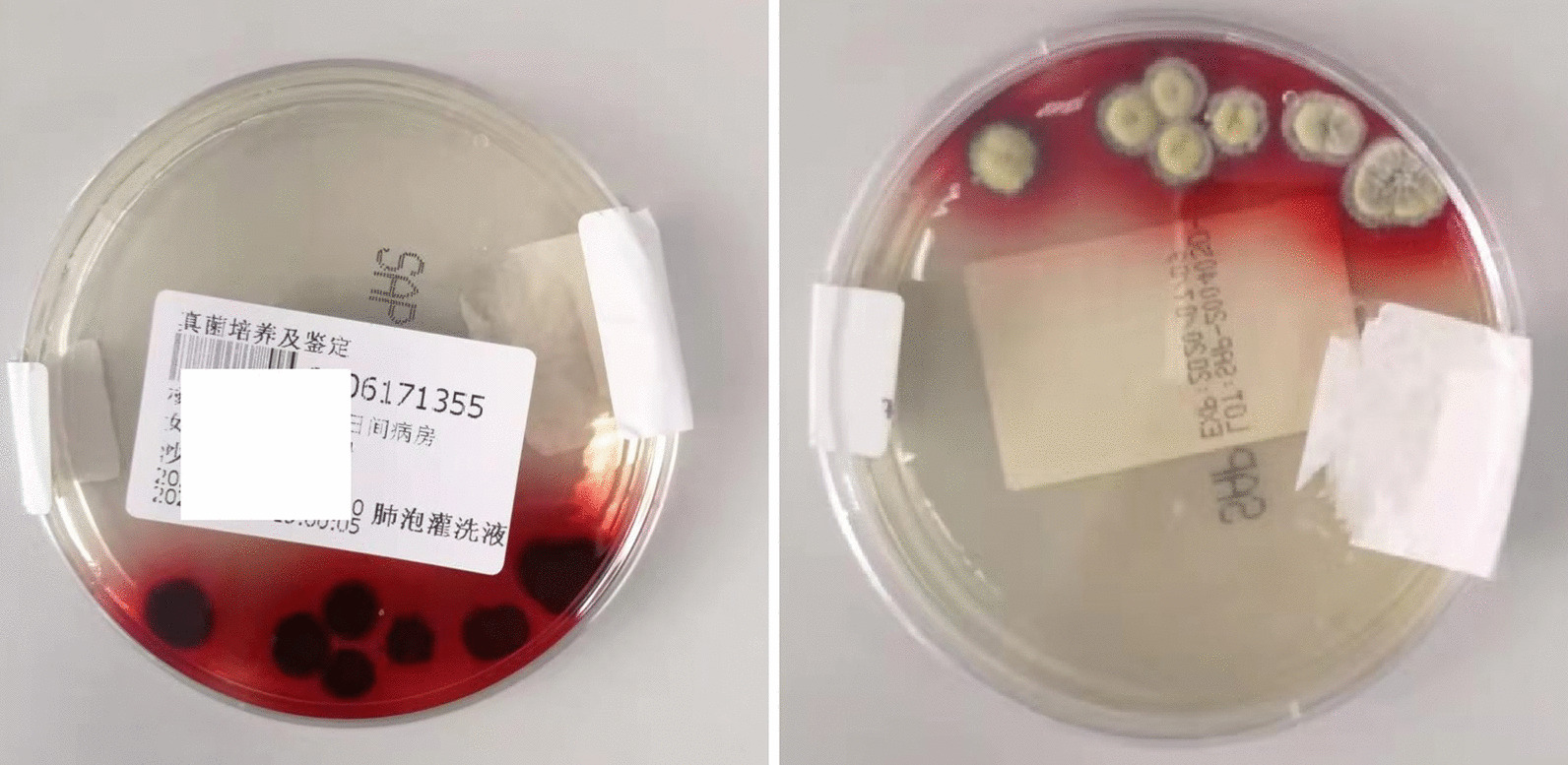
Granular colony of *T. marneffei* with a characteristic soluble red pigment that diffused into the agar after 7 days of incubation at 25 °C in BALF

High-resolution computed tomography (HRCT) showed small airway obstruction lesions, and bilateral diffuse infiltration and local bronchiectasis in both lungs (Fig. 1A–C). Electronic bronchoscopy showed heavy yellow-white purulent secretion in the airway, and bronchoalveolar lavage fluid (BALF) for culture yielded *T. marneffei* (Fig. 2). By the same token, high-throughput sequencing detected *T. marneffei* in BALF. In accordance with pathogenic status, amphotericin B deoxycholate at 20 mg/day was commenced as the primary antifungal therapy for 14 days with good clinical response, and the patient was discharged with oral itraconazole prescribed for 4 weeks.

**Fig. 3:**
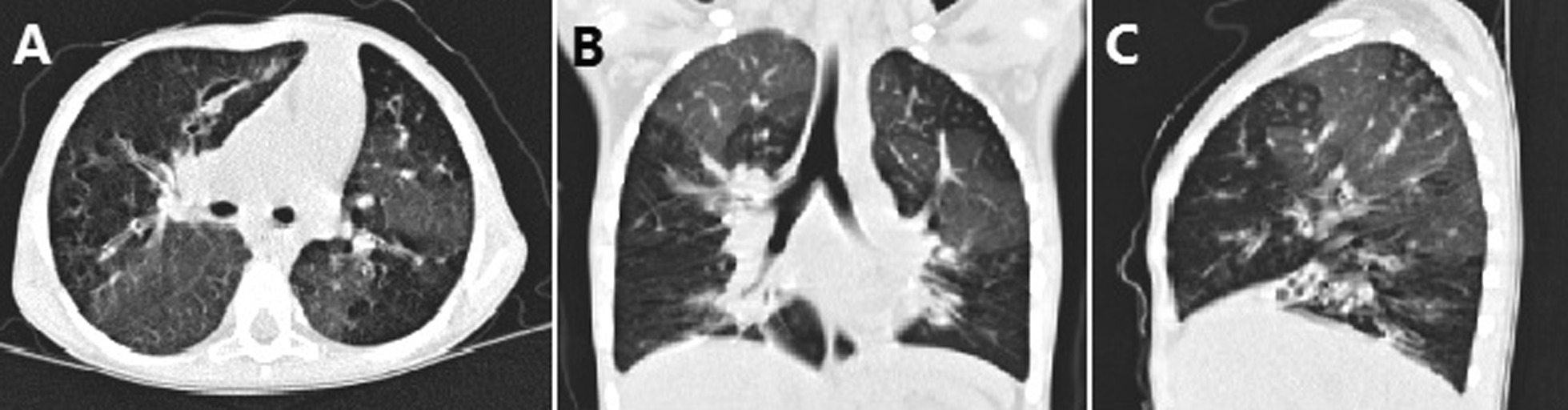
High-resolution CT scan of the chest revealing both lungs scattered widely with ground-glass-like shadows, while focal areas of increased transmittance show “mosaic” changes. Bronchiectasis in lower lobe of both lungs and middle lobe of right lung on second hospitalization

One month after discharge, she presented to the emergency department with shortness of breath and oedema of eyelids and limbs. HRCT showed multiple patchy ground-glass opacities that manifested as mosaic attenuation (Fig. 3A–C). Culture of *T. marneffei* was negative in BALF and blood during this hospitalization. A restrictive abnormality with reduction of diffusion capacity was mainly found in pulmonary function. She was treated with intravenous Ig (400 mg/kg/day) for 3 days as well as aerosol inhalation of budesonide. After 10 days of treatment, the dyspnoea was relieved and she was discharged with recommended continued use of a Symbicort Turbuhaler.

## Follow-up and gene report

**Fig. 4:**
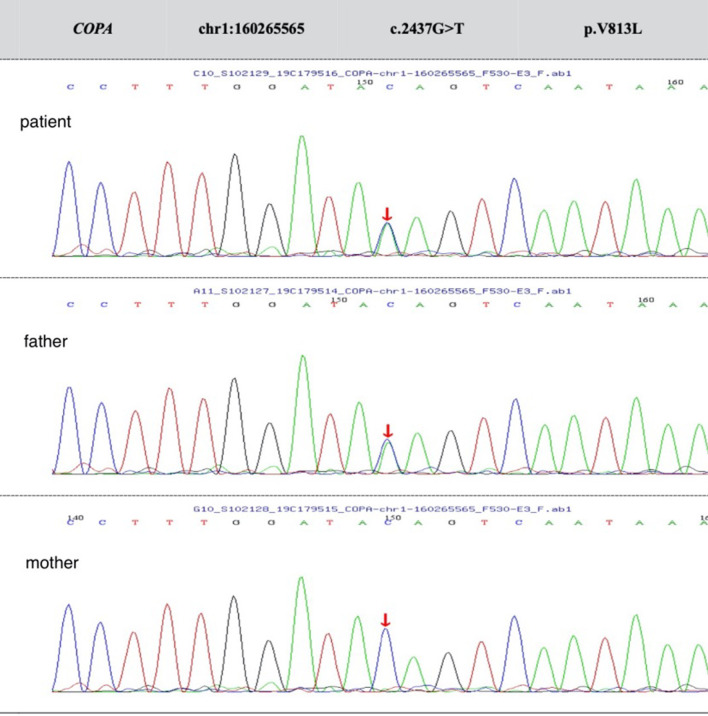
Sequencing analysis of *COPA* in the patient and her father revealed the heterozygous substitution [c.2437G > T, p.V813L ] in chr1:160265565 as a de novo mutation (arrow), and her mother was negative for this mutation. The mutation of nucleotide 2437 from guanine to thymine resulted in the missense mutation of amino acid 813 from valine to leucine

Cough and yellow phlegm were reduced, but intermittent wheezing symptoms still persisted after the patient left hospital. In addition, whole-exome sequencing identified a de novo missense mutation c.2437G > T(p.V813L) in the *COPA* gene (Fig. 4), but the mutation was predicted to be uncertain based on the American Center for Medical Genetics and Genomics guidelines. During the follow-up, the child still wheezed intermittently and did not show any positive symptoms of kidney or autoimmune inflammatory arthritis problems.

## Discussion and conclusion

*T. marneffei* is the only temperature-biphasic pathogenic fungus in *Penicillium*, and is endemic in Southeast Asia [6]. In adults, most *T. marneffei* infections occur in AIDS patients, especially HIV infected, while varying among children. We present the case of a child with sequelae of PIBO arising from *T. marneffei* infection without HIV and accompanied by mutations in the *COPA* gene.

In many aspects, the clinical manifestations of paediatric patients with *T. marneffei* infection are not typical, which is a potential reason for misdiagnosis of *T. marneffei* infection [7]. Our patient presented with fever, cough, and dyspnoea but there was no manifestation of disseminated *T. marneffei* infection, including rash, weight loss, lymphadenopathy, and hepatosplenomegaly in this patient as in the previous reports [8, 9]. Although the clinical history spanned 2 months, the diagnosis of *T. marneffei* infection was not confirmed until she was hospitalized in our centre. Positive culture and high-throughput sequencing of BALF were the most important criteria in the final diagnosis of *T. marneffei* infection in this child, suggesting that BALF can be used for the early diagnosis of such an infection.

PIDs that commonly manifest some degree of hypogammaglobulinemia include selective IgA deficiency, common variable immunodeficiency, and congenital agammaglobulinaemias. Less common causes include agammaglobulinaemia with thymoma (Good syndrome) and X-linked lymphoproliferative syndrome [10]. In addition, concomitant opportunistic infections in this child should raise suspicion of a cellular defect that also affects antibody production, such as nuclear factor κB essential modulator (NEMO; also called IKK-γ) or CD40 ligand (CD154) deficiencies [10, 11]. Because the exact kind of PIDs may be difficult to determine based on the peripheral immunological results alone, genetic testing was carried out. The patient was identified with a *de novo* missense mutation at exon 17 (c.2437G > T, p.V813L) in the *COPA* gene. Patients with *COPA* mutations typically have normal numbers and percentages of lymphocytes and lymphocyte subsets along with unremarkable Ig levels and intact production of specific antibodies [4, 12]. However, the child had markedly decreased Ig with normal numbers of lymphocytes. The exact mechanism by which *COPA* gene mutation causes *T. marneffei* infection is currently unknown.

Pulmonary fungal infections complicated by PIBO sequelae are very rare. Recent research suggests that pulmonary colonization with *Aspergillus* species has been implicated as a potential risk factor in the development of PIBO [13]. However, *T. marneffei* infection with secondary PIBO had not been previously reported. According to her repeated dyspnoea and wheezing over a period of longer than 2 months and mosaic signs on HRCT, despite lung biopsy being essential for the diagnosis of PIBO this procedure was not performed in this patient because of her tender age, although PIBO was also considered in the differential diagnosis. Interestingly, lung involvement is usually in the form of interstitial lung disease in patients with *COPA* gene mutation [14] and, as such, the mechanism of PIBO might be a combination of *T. marneffei* infection and *COPA* gene mutation.

In conclusion, it must be stressed that while *T. marneffei* infection with PIBO is very rare, this patient also showed a de novo missense mutation in the *COPA* gene. Evidence from this report suggests that all clinicians must consider PIBO as a possible sequela in an HIV-negative paediatric patient with *T. marneffei* infection. Moreover, the role of *COPA* in *T. marneffei* infection is worthy of further study.

## Data Availability

All data and materials of this article are included in the manuscript and are thus available to the reader.

## Abbreviations

AIDS: Immunodeficiency syndrome
ACMG: American Center for Medical Genetics and Genomics
BALF: Bronchoalveolar lavage fluid
HIV: Human immunodeficiency virus
HRCT: High-resolution computed tomography
NBT: Nitroblue tetrazolium test
NK: Natural killer
PIBO: Post-infectious bronchiolitis obliterans
PID: Primary immunodeficiency disease
